# A multidisciplinary study of pain in cats undergoing dental extractions: A prospective, blinded, clinical trial

**DOI:** 10.1371/journal.pone.0213195

**Published:** 2019-03-01

**Authors:** Ryota Watanabe, Graeme Doodnaught, Caroline Proulx, Jean-Philippe Auger, Beatriz Monteiro, Yvan Dumais, Guy Beauchamp, Mariela Segura, Paulo Steagall

**Affiliations:** 1 Département de sciences cliniques, Faculté de médecine vétérinaire, Université de Montréal, Saint-Hyacinthe, Quebec, Canada; 2 Groupe de recherche sur les maladies infectieuses en production animale (GREMIP) et Département de pathologie et microbiologie, Faculté de médecine vétérinaire, Université de Montréal, Saint-Hyacinthe, Quebec, Canada; 3 Département de biomédecine vétérinaire, Faculté de médecine vétérinaire, Université de Montréal, Saint-Hyacinthe, Quebec, Canada; University of Bari, ITALY

## Abstract

This study aimed to evaluate pain scores, analgesic requirements, food intake and serum inflammatory cytokines in cats before and after clinically recommended dental treatment. Twenty-four cats were included in a prospective, blinded clinical trial. Cats were equally divided into minimal (minimal dental treatment) or severe (multiple dental extractions) oral disease groups. They were admitted (day 0) and underwent oral examination/radiographs/treatment under general anesthesia (day 1; acepromazine-hydromorphone-propofol-isoflurane-meloxicam-local anesthetic blocks). Serum inflammatory cytokines were measured on days 0 and 6. Pain was scored using the Glasgow composite measure pain scale-feline (CMPS-F). Rescue analgesia was administered with hydromorphone if CMPS-F ≥ 5/20. Dry and soft food intake (%) during 3 minutes and 2 hours, and daily soft food were calculated. The Cochran-Mantel-Haenszel and Chi-square tests, Spearman’s rank correlation and linear mixed models were used for statistical analysis (alpha = 0.05). Pain scores were significantly increased in cats with severe disease when compared with baseline (up to day 4) and minimal disease (all postoperative time points). Prevalence of rescue analgesia was significantly higher in severe (91.7%) than minimal disease (0%); analgesics were required up to day 3. Pain scores and frequency of rescue analgesia were significantly correlated with the number of tooth extractions, gingival and calculus index. Prevalence of rescue analgesia was significantly correlated with the number of missing teeth, teeth extractions and gingival index. Dry and soft food intake during 3 minutes, and dry food intake during 2 hours were significantly lower in the severe than minimal disease group throughout the study. Some cytokines differed between groups between day 0 and day 6 and were associated with the presence of tooth resorption and number of missing tooth and tooth fractures. Long-term analgesia is required after dental extractions in cats with severe oral disease. This condition reduces food intake and influences serum inflammatory cytokines.

## Introduction

Pain is a serious welfare issue that produces long-term distress with significant deleterious effects affecting quality of life (QoL) in humans [1–4]. Periodontal disease including gingivitis and periodontitis, is one of the most commonly reported diseases in humans and companion animals [3,5–8]. In cats, it produces pain, inflammation, dysphagia, halitosis, weight loss and oral hemorrhage; aggressive full-mouth extractions are commonly required as treatment [9,10]. Nevertheless, pain scores and analgesic requirements have not been systematically investigated in cats with oral disease undergoing dental extractions. It is unknown how oral treatment can affect soft and dry food intake perioperatively which could significantly impact the nutritional status of these patients.

Gram-negative bacteria are the major pathogen of periodontal disease. They release endotoxins (lipopolysaccharides; LPS) that mediate the release of inflammatory cytokines [e.g. interleukin (IL)-1, IL-6, tumor necrosis factor (TNF)] which are strongly correlated with the progression of the disease in humans [11–14]. In veterinary medicine, local inflammatory cytokines have been evaluated in dental resorptive lesion and bone in cats with periodontal disease [15–17]. However, serum concentrations of inflammatory cytokines in cats with periodontal disease have not been studied. The study of serum inflammatory cytokines could provide valuable insight in the pathogenesis of oral disease in cats.

The objectives of this study were to evaluate the effects of oral disease and its treatment on pain scores, analgesic requirements, food intake and serum inflammatory cytokines in cats with severe or minimal oral disease. The hypotheses were that cats with severe disease would have higher pain scores and analgesic requirements and reduced food intake than those with minimal disease before and after dental extractions, and that concentrations of serum inflammatory cytokines would differ between groups.

## Materials and methods

### Study design

This study was approved by the Institutional Animal Care and Use Committee of the Université de Montréal (protocol 17-Rech-1890) and is reported according to the CONSORT guidelines [18]. The experimental study was performed at the Centre hospitalier universitaire vétérinaire (CHUV), the veterinary teaching hospital of the Faculty of Veterinary Medicine of the Université de Montréal, between July 2017 and February 2018. The study design was a prospective, blinded, controlled clinical trial. Randomization was not feasible because cats were allocated to one of two groups according to their disease severity (severely versus minimally affected cats).

### Animals

**Fig 1:**
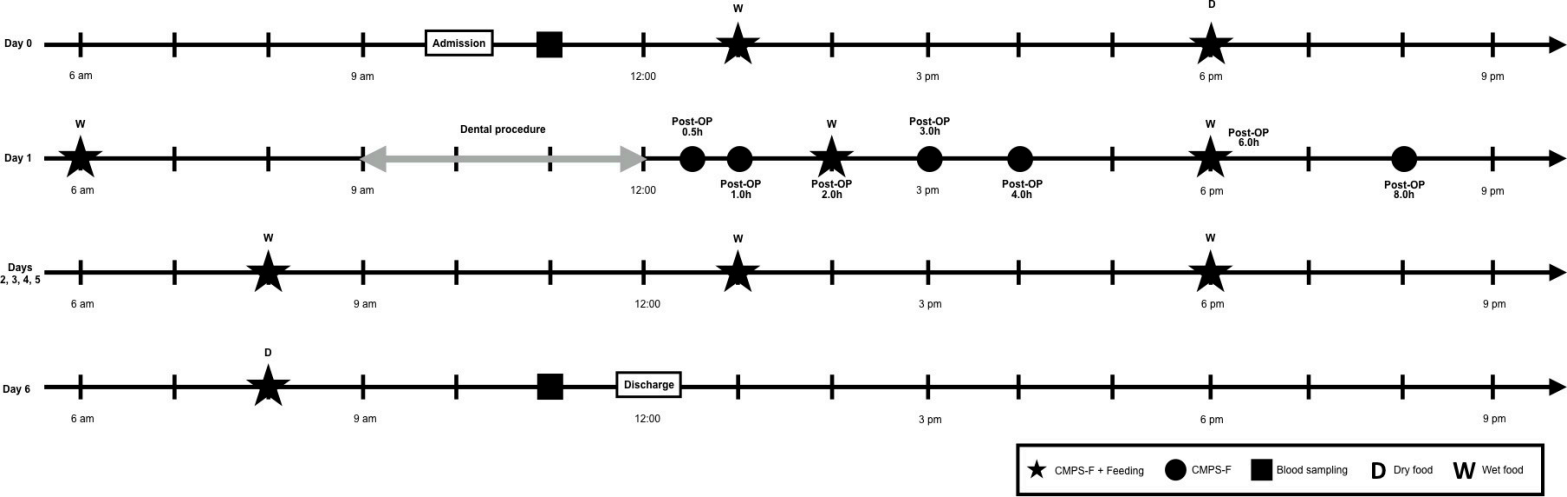
Schematic of time points for assessment of pain and food intake in an example of a dental procedure of 3 hours. CMPS-F: Glasgow composite measure pain scale-feline.

Twenty-four adult (> 1 year of age) cats of different breeds and gender with naturally-occurring oral disease were studied. Cats were recruited by the investigators (PS and BM) from shelter facilities after informed written consent based on the severity of oral disease. Initial oral examination was performed by the local shelter veterinarians who were aware of the presence or absence of clinical signs related to dental disease. They were admitted approximately 24 hours before general anesthesia (day 0) for dental treatment which was performed on day 1. Cats were discharged on day 6 (7 days after arrival and 6 days after treatment of oral disease) (Fig 1). Animals were housed in stainless steel cages in a cat only ward with access to water *ad libitum*, toys, litter box and bedding. A cardboard box was also provided offering additional shelter and an elevated surface. Environmental enrichment was provided following the guidelines of the American Association of Feline Practitioners [19].

### Inclusion and exclusion criteria

Cats with body condition score between 4–6 out of 9, and with minimal or severe oral disease were included [20]. Inclusion was also based on history, medical records, complete physical examination, and hematology and biochemical panel. Feral cats were excluded. Cats were also excluded if they had concurrent medical conditions, systemic disorders (e.g. cancer, renal, cardiovascular, hepatic, or gastrointestinal disease) and/or received any medication including analgesics and antibiotics for up to 10 days before the study had begun.

### Treatment of oral disease

#### Group allocation

Complete dental examination and radiography were performed, and patients underwent dental cleaning and dental extractions (if needed) by a board-certified dentist (YD) and a resident (CP) of the American Veterinary Dental College (AVDC). Staging of periodontal disease was based on dental examination, radiography, gingival index, calculus index, number of teeth resorbed, and fractured and/or missing as defined by the AVDC [21]. Group allocation (minimal or severe) was determined upon recruitment after oral examination and confirmed according to a scoring system based on the type and number of extracted teeth: canine tooth—3 points; third premolar of maxilla or molar of mandible—2 points, second premolar of maxilla or premolar of mandible—1 point. A score of 2 points was given if seven or more incisive teeth and/or first premolars of the mandible were extracted; otherwise a score of 1 point was given if six or fewer teeth were removed. The total dental score was calculated, and cats were allocated to the minimal disease group if dental score ≤ 7, and to the severe disease group if dental score was ≥ 8. This cut-off was determined based on the expected level of pain that would be clinically significant in cats with score ≥ 8.

#### Anesthesia and analgesic protocol

All cats were premedicated with an intramuscular (IM) injection of acepromazine (0.02 mg kg^-1^; Acepromazine maleate, Gentès & Bolduc, Saint-Hyacinthe, QC, Canada) and hydromorphone (0.1 mg kg^-1^; Hydromorphone hydrochloride 2 mg mL^-1^, Sandoz, Boucherville, QC, Canada). A eutectic mixture of local anesthetic cream (EMLA cream lidocaine 2.5% and procaine 2.5% cream, Astra Zeneca, Mississauga, ON, Canada) was applied to the skin over the cephalic vein after clipping and covered with plastic film and adhesive bandage. Approximately 20 minutes later, a 22-G intravenous (IV) catheter was aseptically placed in the cephalic vein and induction of anesthesia was performed with propofol (Propoflo 28, Zoetis, Kirkland, QC, Canada) administered IV to effect (4.0 ± 1.2 mg kg^-1^). After spraying the arytenoid cartilages with 0.05 mL of lidocaine 2% (Lidocaine hydrochloride sterile injection, 20 mg mL^-1^, Vétoquinol N.-A.Inc, Lavaltrie, QC, Canada), cats were intubated with an appropriately sized cuffed endotracheal tube and connected to a coaxial Mapleson D system. Anesthesia was maintained with isoflurane (Isoflurane USP, Fresenius Kabi, Toronto, ON, Canada) carried in 100% oxygen. Monitoring was performed with a multiparametric monitor (Lifewindow 6000V Veterinary Multiparameter Monitor; Digicare Animal Health, Boynton Beach, FL, USA) including pulse oximetry, electrocardiography, capnography, inspired and expired concentrations of isoflurane, indirect blood pressure via oscillometry, and rectal temperature probe. Blood pressure was also monitored with a Doppler flow monitor and a sphygmomanometer [22,23]. The cuff width used for blood pressure monitoring was approximately 40% of the limb circumference. A balanced crystalloid solution was administered (2–5 ml kg^-1^ hour^-1^) based on patient needs throughout the anesthetic period. Cats received local anesthetic blocks with bupivacaine 0.5% (Sensorcaine, AstraZeneca, ON, Canada) using a 25-G needle based on the anatomical sites of dental extraction(s) including the mental, infraorbital, maxillary and/or inferior alveolar mandibular nerve blocks approximately 20 minutes before teeth extraction. The total dose of bupivacaine for all anesthetic blocks was up to 2 mg kg^-1^. Meloxicam (0.2 mg kg^-1^, subcutaneously, Metacam 5 mg mL^-1^ Solution for Injection; Boehringer Ingelheim, Burlington, ON, Canada) was administered at the end of the surgical procedure. Three additional doses of meloxicam (0.05 mg kg^-1^, Metacam 0.5mg mL^-1^ Oral Suspension for Cats; Boehringer Ingelheim, Burlington, ON, Canada) were administered orally at 24, 48 and 72 hours after the first dose according to label recommendations in Canada.

### Pain assessment

Pain assessment was performed by a trained observer [RW] who was blinded to the disease severity using the Glasgow composite measure pain scale-feline (CMPS-F) [24]. Pain was evaluated preoperatively (at 1 pm and 6 pm on day 0, approximately three hours before the dental procedure on day 1, and at 0.5, 1, 2, 3, 4, 6, 8 hours after the end of anesthesia on day 1. Pain was also assessed at 8 am, 1 pm and 6 pm on days 2, 3, 4, 5 and again at 8 am on day 6 according to Fig 1. Baseline pain scores were calculated using the mean of three preoperative values. Rescue analgesia was administered if CMPS-F scores were ≥ 5/20 with hydromorphone either at 0.05 mg kg^-1^ IV (if the intravenous catheter was in place, first 24 hours after surgery) or 0.1 mg kg^-1^ IM (if the intravenous catheter had been removed). In this case, pain was reassessed 30 minutes later to ensure the patient’s comfort. Based on previous literature on the duration of hydromorphone in cats, pain scores obtained within 2 hours of IV and within 6 hours of IM injection of rescue analgesia were excluded from statistical analysis. [25,26]. However, pain assessment was performed systematically until the end of the study period.

### Food intake

All cats were fed a commercially available dry food (Hill’s Science Diet, Adult Optimal Care–Dry; Hill’s Pet Nutrition Canada Inc., Mississauga, ON, Canada) on days 0 and 6. A commercial canned prescription recovery diet (Hill’s Prescription Diet a/d; Hill’s Pet Nutrition Canada Inc., Mississauga, ON, Canada) was provided at all other time points during the study (Fig 1). Total amount (100%) of food offered per day was calculated based on the following equation (kcal): 70 × body weight (kg)^0.75^ [27]. Cats were served 33.3% of their daily total amount at each time point (Fig 1). Food intake (percentage of the total amount offered) during 3 minutes and 2 hours was calculated (except on the morning of day 1 when cats were fed for only 3 minutes) for each time point; any remaining food was removed after 2 hours. Daily food intake (percentage of the total amount offered) was calculated using the mean of three meals offered per day. Baseline food intake (%) was calculated using the mean of the two preoperative soft food meals. Food intake obtained within 2 hours of IV and within 6 hours of IM injection of rescue analgesia were excluded from statistical analysis, but assessments were continued until the end of the study.

### Inflammatory cytokines

#### Sample collection

Whole blood was collected via venipuncture using the jugular vein on days 0 (before the first pain and food intake assessments) and 6 (after final pain and food intake assessments) (Fig 1), placed into a sterile 3 mL anticoagulant-free glass tube (Monoject Blood Collection Tube; Covidien Canada, Saint-Laurent, QC, Canada) and allowed to clot at room temperature for 40 minutes. Clotted samples were then centrifuged at 3000 rpm for 10 minutes, and serum was removed, aliquoted, and stored in cryovials at -80°C until final analyses [28].

#### Evaluation of serum concentration

Samples were warmed to room temperature and analyzed for concentrations of 19 analytes using commercially available feline-specific multiplex cytokine kits (FCYTMAG-20K-PMX, Luminex Corporation, TX, USA). The kit was used according to the manufacturer’s recommendations. The plate was analyzed using a dedicated reader (MAGPIX, Luminex Corporation, TX, USA) and software (xPONENT v.4.2, Luminex Corporation, TX, USA). The quality control samples, standard curves, and bead counts were assessed and conformed to manufacturer recommendations. Analytes with concentrations more than 50% out of the range of analysis were excluded from the analyses.

### Statistical analyses

Statistical analyses were performed using standard statistical software (SAS version 9.3; SAS Institute, Cary, NC, USA). Power analysis revealed that this study needed 8 cats per group to detect a difference of three points in the CMPS-F pain scores between the two groups 80% of the time using an alpha value of 0.05, and a standard deviation within group of 2 points. These values were based on clinical experience where changes in three points in CMPS-F were clinically relevant. Therefore, the authors decided to include 12 cats per group to assure adequate power considering the individual variability of oral disease in cats. Data were tested for normality using a Shapiro-Wilk test. Demographic data for each treatment group were compared using two-sample t-tests or Mann-Whitney U tests where appropriate. The CMPS-F ordinal scores were compared between baseline and each time point, and between dental severity groups at each time point using Cochran-Mantel-Haenszel statistics. Prevalence of rescue analgesia between groups was compared using the exact chi-square test. Serum concentration of inflammatory cytokines were compared after log_10_ transformation between days 0 and 6, and between dental severity groups using a linear mixed model followed by contrasts between pairs of means using the Benjamini-Hochberg sequential alpha adjustment procedure. Food intake was compared with baseline and between dental severity groups using a linear mixed model with the same contrast comparisons. Correlation between pain scores and the frequency and prevalence of rescue analgesia, and periodontal staging, gingival index, calculus index, number of tooth resorption, tooth fracture and missing were evaluated using Spearman’s correlation. The alpha level was set at 5% throughout.

## Results

Descriptive statistics for age, body weight, body condition score, surgery (time elapsed from the first scaling until the end of scaling or placement of the last suture) and anesthesia (time elapsed from induction of propofol to turning off the vaporizer dial) times, and dental score and number of extracted teeth are presented in Table 1. Cats in the minimal disease group were typically younger and lighter and required less time for surgery and anesthesia than those in the severe group (Table 1). One cat from the minimal disease group was excluded because of wound dehiscence in the postoperative period requiring further treatment. Therefore, only preoperative data of this cat was included in the analysis.

**Table 1:** Demographic data, surgery and anesthesia times in cats with minimal and severe oral disease.

Variable	Minimal (n = 12)	Severe (n = 12)	*p* value
Age (years)	3.6 (2.0)	8.5 (2.2)	< 0.0001
Body weight (kg)	4.0 (0.6)	5.8 (1.9)	0.007
Body condition score (1–9)	5 (5–6)	6 (4–6)	0.078
Surgery time (minutes)	98.8 (47.4)	261.0 (72.2)	< 0.0001
Anesthesia time (minutes)	103.8 (48.2)	274.7 (70.3)	< 0.0001
Dental score	1 (0–4)	17 (8–28)	< 0.0001
Number of extracted teeth	2 (0–5)	17 (8–30)	< 0.0001

Values are expressed as mean (SD) with exception of body condition score which is reported as median (min-max).

### Pain assessment

Pain scores in each group are shown in Table 2. In the severe group, CMPS-F scores were significantly higher at 1, 2, 3, 4, 6 and 8 hours on day 1, at all three time points on day 2 and at 1 pm on days 3 and 4 when compared with baseline. In the minimal group, there were no significant differences between baseline and any postoperative time point (*p* = 0.13). CMPS-F scores in the severe group were significantly higher than the minimal group at all postoperative time points. Rescue analgesia was administered to 11 cats in the severe group (91.7%) and to none in the minimal group (0%) (*p* < 0.0001) (Table 3). CMPS-F scores and the frequency of rescue analgesia were significantly correlated with number of tooth extractions (*r* = 0.84, *p* = 0.0001 and *r* = 0.83, *p* = < 0.0001; respectively), gingival index (*r* = 0.70, *p* = 0.001 and *r* = 0.67, *p* = 0.003; respectively) and calculus index (*r* = 0.48, *p* = 0.02, and *r* = 0.47, *p* = 0.03; respectively). Prevalence of rescue analgesia was significantly correlated with the number of missing teeth and tooth extractions, gingival index and calculus index (*r* = 0.46, *p* = 0.03; *r* = 0.78, *p* = < 0.0001; *r* = 0.72, *p* = < 0.0001 and *r* = 0.56, *p* = 0.006; respectively).

**Table 2:** Median (min-max) of pain scores using the Glasgow Composite Pain Scale Feline (CMPS-F) in cats with minimal or severe oral disease undergoing dental extractions throughout the study.

Time point		Minimal	Severe		*p* value between groups
		CMPS-F scores	CMPS-F scores	*p* value comparisons with baseline (severe group only)	
Baseline		0 (0)	0 (0–2)		0.083
Day 1 (Postoperative)	0.5 hours	0 (0)	0.5 (0–4)	0.12	0.020
	1 hours	0 (0)	1 (0–6)	0.042	0.017
	2 hours	0 (0)	2 (0–5)	0.016	0.0006
	3 hours	0 (0–1)	3 (1–7)	0.016	0.0011
	4 hours	0 (0–1)	5 (0–7)	0.029	0.0011
	6 hours	0 (0)	2 (1–6)	0.039	0.0013
	8 hours	0 (0)	1.5 (1–5)	0.027	0.0015
Day 2	8 am	0 (0–1)	2 (0–5)	0.008	0.0005
	1 pm	0 (0–1)	2 (1–6)	0.013	0.0008
	6 pm	0 (0–3)	2 (0–7)	0.039	0.0150
Day 3	8 am	0 (0)	1 (0–5)	0.053	0.0038
	1 pm	0 (0)	2 (0–2)	0.016	0.0001
	6 pm	0 (0)	1 (0–6)	0.052	0.0056
Day 4	8 am	0 (0)	1 (0–4)	0.096	0.0036
	1 pm	0 (0)	1.5 (0.4)	0.043	0.0028
	6 pm	0 (0)	1 (0–3)	0.061	0.0009
Day 5	8 am	0 (0)	1 (0–2)	0.083	0.0026
	1 pm	0 (0)	0.5 (0–1)	0.74	0.0076
	6 pm	0 (0)	0.5 (0–2)	0.32	0.013
Day 6		0 (0)	1 (0–2)	0.25	0.0062

**Table 3:** Number of cats receiving rescue analgesia at each time point during the study.

Group	Day 1 (postoperative)							Day 2	Day 3	Days 4, 5, 6	Total	*p* value
	0.5 h	1 h	2 h	3 h	4 h	6 h	8 h					
Minimal	0	0	0	0	0	0	0	0	0	0	0 (0%)	< 0.0001
Severe	0	2	1	2	5	2	2	5	2	0	21 (91.7%)	

### Food intake

#### Soft food intake

In the severe group, soft food intake during 3 minutes and daily soft food intake during 3 minutes were significantly lower than the minimal group throughout the study (Table 4). When compared with baseline, food intake was significantly higher in the minimal group at 6 hours after the end of anesthesia and significantly lower in the severe group in the morning of day 4. Soft food intake during 2 hours and daily soft food intake during 2 hours were not significantly different between groups.

**Table 4:** Mean (SD) of dry and soft food intake (%) in cats with minimal or severe oral disease undergoing dental treatment.

Time point		Food intake (%) during 3 minutes			Food intake (%) during 2 hours			*p* value compared with baseline (3 minutes)		*p* value compared with baseline (2 hours)	
		Minimal	Severe	*p* value between groups	Minimal	Severe	*p* value between groups	Minimal	Severe	Minimal	Severe
Baseline	Soft food	63.7 (9.0)	42.4 (7.9)	0.0538	94.3 (4.8)	85.1 (9.2)	0.44				
	Dry food	77.1 (6.0)	28.1 (6.1)	0.0001	94.9 (5.1)	63.6 (11.5)	0.012				
Day 1 (Postoperative) Soft food	2 hours	67.4 (12.3)	29.2 (8.3)	0.0002	87.3 (9.40)	86.1 (10.5)	0.99	0.26	0.062	0.45	0.86
	6 hours	83.6 (7.8)	33.2 (10.4)	< 0.0001	100 (0.0)	72.7 (18.9)	0.002*	0.001	0.028*	0.45	0.025*
	Daily food intake	75.5 (9.5)	27.7 (27.7)	< 0.0001	93.6 (4.7)	52.1 (48.0)	0.003*	0.020*	0.049*	0.95	0.23
Day 2	8 am	59.7 (9.5)	35.4 (7.1)	0.0102*	90.9 (9.1)	75.5 (11.6)	0.092	0.80	0.17	0.77	0.14
Soft food	1 pm	66.2 (8.0)	40.1 (8.0)	0.0097*	93.8 (4.2)	77.2 (10.1)	0.11	0.62	0.52	0.94	0.24
	6 pm	66.9 (8.8)	37.1 (3.5)	0.0041*	92.4 (7.6)	85.2 (6.6)	0.25	0.46	0.40	0.99	0.61
	Daily food intake	64.3 (7.8)	36.0 (5.1)	0.004	92.4 (5.5)	72.6 (7.2)	0.020*	0.75	0.90	0.68	0.78
Day 3	8 am	60.7 (8.8)	29.8 (5.0)	0.0057	93.4 (6.6)	89.4 (5.2)	0.35	0.65	0.057	0.94	0.87
Soft food	1 pm	68.3 (7.6)	31.6 (5.0)	0.0017*	100 (0.0)	74.2 (11.0)	0.0155*	0.40	0.21	0.94	0.17
	6 pm	76.1 (9.5)	33.1 (4.0)	0.0001	90.9 (9.1)	82.2 (9.0)	0.28	0.052	0.19	0.99	0.47
	Daily food intake	68.4 (7.9)	31.5 (4.0)	0.0007	94.8 (5.2)	82.0 (5.1)	0.020*	0.79	0.67	0.73	0.24
Day 4	8 am	57.5 (9.6)	16.7 (5.0)	0.0004	91.9 (8.1)	81.0 (9.2)	0.16	0.39	0.0003	0.95	0.38
Soft food	1 pm	70.0 (6.8)	38.7 (5.7)	0.0129*	100 (0)	94.8 (5.3)	0.63	0.41	0.91	0.45	0.26
	6 pm	74.0 (8.2)	22.2 (4.5)	< 0.0001	91.9 (8.1)	73.6 (9.3)	0.044*	0.094	0.0076*	0.95	0.10
	Daily food intake	67.2 (7.4)	25.9 (4.2)	0.0002	94.6 (5.4)	83.1 (5.8)	0.045*	0.98	0.17	0.75	0.09
Day 5	8 am	70.4 (9.2)	22.3 (5.4)	< 0.0001	93.4 (6.6)	81.8 (10.5)	0.25	0.27	0.004*	0.94	0.68
Soft food	1 pm	73.2 (8.7)	38.0 (3.7)	0.0025*	100 (0.0)	92.0 (5.8)	0.43	0.097	0.86	0.45	0.45
	6 pm	75.4 (9.0)	29.9 (4.7)	0.0002	91.9 (8.1)	78.9 (9.9)	0.19	0.09	0.18	0.95	0.43
	Daily food intake	73.0 (8.4)	30.0 (3.9)	< 0.0001	95.1 (4.9)	84.3 (7.5)	0.16	0.27	0.54	0.70	0.007*
Day 6 Dry food	8 am	71.2 (8.2)	18.6 (5.5)	< 0.0001	91.9 (8.1)	57.7 (11.4)	0.009	0.48	0.19	0.84	0.68

* not significant after adjustment.

#### Dry food intake

Dry food intake during 3 minutes and 2 hours was significantly lower in the severe group when compared with the minimal group at days 0 and 6 (Table 4).

### Inflammatory cytokines

GM-CSF was excluded from statistical analyses because more than 50% of concentrations were beyond the reference range. Interferon (IFN)-γ, IL-4, IL-6, IL-8, regulated on activation-normal T cell expressed and secreted (RANTES), stem cell factor (SCF), and monocyte chemoattractant protein (MCP) -1 were significantly lower on day 6 than on day 0 in cats with severe oral disease (Table 5). The concentrations of SCF were significantly higher in cats with severe than those with minimal disease on day 0. IL-12p40 was significantly higher on day 6 than on day 0 in both groups. There were positive associations between soluble FAS (sFAS), IL-6, stromal cell-derived factor (SDF) -1, and MCP-1, and the number of teeth resorption (*p* = 0.048, 0.028, 0.012, and 0.047, respectively), between sFAS and the number of missing teeth (*p* = 0.02), between keratinocyte chemoattractant (KC) and the number of teeth fracture (*p* = 0.038), and a negative association between sFAS and TNF-α and the number of teeth fracture (*p* = 0.03 and 0.011, respectively).

**Table 5:** Log_10_ transformed least squares means (SEM) for serum concentrations of inflammatory cytokines and chemokines in cats with minimal or several oral disease undergoing dental treatment.

Analyte	Minimal			Severe			*p* value between groups at day 0	*p* value between groups at day 6
	Day 0	Day 6	*p* value between days 0 and 6	Day 0	Day 6	*p* value between days 0 and 6		
sFAS	0.82 (0.17)	0.74 (0.17)	0.33	0.78 (0.17)	0.66 (0.17)	0.12	0.87	0.75
FLT-3L	1.79 (0.06)	1.81 (0.06)	0.52	1.79 (0.06)	1.79 (0.06)	0.95	0.93	0.76
IFN-γ	1.89 (0.14)	1.87 (0.14)	0.63	2.28 (0.14)	2.16 (0.14)	0.008	0.07	0.17
IL-1β	1.17 (0.32)	1.21 (0.32)	0.66	1.56 (0.25)	1.39 (0.25)	0.022*	0.37	0.68
IL-2	1.21 (0.22)	1.03 (0.22)	0.08	1.47 (0.22)	1.41 (0.22)	0.53	0.43	0.24
IL-4	2.04 (0.17)	1.99 (0.17)	0.28	2.57 (0.17)	2.41 (0.17)	0.0004	0.04*	0.10
IL-6	1.95 (0.15)	1.91 (0.15)	0.28	2.07 (0.15)	1.93 (0.15)	0.0006	0.94	0.94
IL-8	1.39 (0.09)	1.24 (0.09)	0.003	1.59 (0.09)	1.46 (0.09)	0.006	0.11	0.08
IL-12p40	2.52 (0.08)	2.62 (0.08)	0.005	2.52 (0.08)	2.66 (0.08)	0.0002	0.99	0.72
IL-13	1.35 (0.15)	1.28 (0.15)	0.11	1.34 (0.14)	1.26 (0.14)	0.03*	0.97	0.90
IL-18	2.00 (0.10)	2.02 (0.11)	0.71	2.12 (0.10)	1.99 (0.10)	0.031*	0.41	0.81
KC	0.68 (0.32)	0.89 (0.33)	0.49	1.02 (0.28)	1.58 (0.26)	0.033*	0.44	0.13
MCP-1	2.89 (0.13)	2.82 (0.13)	0.11	3.07 (0.13)	2.97 (0.13)	0.007	0.48	0.48
PDGF-BB	2.76 (0.13)	2.77 (0.13)	0.93	2.98 (0.14)	2.75 (0.14)	0.015*	0.28	0.94
RANTES	1.51 (0.07)	1.48 (0.07)	0.44	1.54 (0.07)	1.43 (0.07)	0.003	0.75	0.58
SCF	2.07 (0.07)	2.05 (0.07)	0.69	2.35 (0.07)	2.22 (0.07)	0.0003	0.10	0.006
SDF-1	2.86 (0.09)	2.91 (0.09)	0.39	2.91 (0.10)	2.84 (0.10)	0.85	0.77	0.61
TNF-α	1.40 (0.45)	1.50 (0.45)	0.57	2.24 (0.39)	1.97 (0.39)	0.11	0.21	0.48

* not significant after adjustment.

## Discussion

This study showed that cats with severe oral disease undergoing dental treatment had significantly higher postoperative pain scores and analgesic requirements, and significantly lower soft and dry food intake when compared with those with minimal oral disease. Additionally, pain scores and frequency and prevalence of rescue analgesia were correlated with some dental parameters and specific serum inflammatory cytokines.

Postoperative pain scores in the severe group were significantly higher than baseline up to day 4 and throughout the study when compared with the minimal disease group. These findings suggest that not only individuals with severe oral disease have discomfort and pain before surgery but also that multiple dental extractions produce severe pain despite the administration of multimodal analgesia including local anesthetic blocks, a non-steroidal anti-inflammatory drug and an opioid. This study also suggests that cats should be hospitalized after multiple dental extractions for appropriate pain management. These patients require the administration of opioids for pain relief up to 72 hours after surgery based on the high prevalence of postoperative rescue analgesia in the severe group (91.7%). Their pain scores are still higher than those cats with minimal oral disease up to 6 days after surgery. There is a clear need for long-term analgesia and better analgesic treatments to address these patients’ needs. Pain scores and frequency and prevalence of rescue analgesia were correlated with specific dental parameters (i.e. number of tooth extractions, gingival and calculus index). Based on these findings, postoperative analgesic requirements might be predicted based on intraoperative oral examination and number of extractions and missing teeth. Oral disease has been considered a welfare issue in the guidelines of the World Small Animal Association Dental Standardization Committee with a negative impact in the quality of life of companion animals [29]. Indeed, it produces severe pain and inflammation before and after treatment in cats [30]. Future studies are warranted to determine oral disease pain-induced behaviors that would facilitate feline pain recognition and assessment in clinical practice. Treatment outcomes could improve with better identification of pain behaviors by veterinarians, technicians and even cat owners.

With the exception of day 1, food was withdrawn two hours after feeding which could underestimate actual daily food intake. The 2-hour interval was chosen to minimize bias over the next food intake evaluation especially for those cats that prefer fresh meals. Soft and dry food intake during 3 minutes, and dry food intake during two hours were significantly decreased in the severe disease group compared with those with minimal disease. This finding indicates that cats with severe disease may take longer to eat before and after dental extractions than cats with less severe disease. Additionally, this study showed that dry food intake might induce pain in cats with severe oral disease since these changes were observed before dental extractions. This would further compromise feline welfare and quality of life of these patients. Nutritional assessment via time taken for soft and dry food intake could be a useful indicator of oral pain and used in clinical studies. This study suggests that soft food should be offered to cats after multiple dental extractions for at least one week after surgery.

The concentrations of serum inflammatory cytokines in cats with oral disease were evaluated herein. However, other concomitant inflammatory conditions could have influenced our results. For example, degenerative joint disease (DJD) is a common disease in cats that causes inflammation and increases the concentrations of inflammatory biomarkers [28]. In this clinical trial, the authors did not specifically investigate the presence or absence of DJD as part of our inclusion/exclusion criterion. Therefore, it is possible that some cats enrolled into the study had DJD and results could have been biased [28]. Additionally, cats received a nonsteroidal anti-inflammatory drug until day 4 as part of multimodal analgesia. It is not known how meloxicam may have affected the concentrations of inflammatory cytokines. Nonetheless, baseline values should not have been affected since treatments were giving intra- and postoperatively. The pharmacokinetics of meloxicam have been described in cats [31] and the concentration of these biomarkers should not have been influenced by day 6 (two days after the last dose of the drug). Finally, both groups received the same dosage regimens and for the purpose of this study, results are comparable. In humans, concentrations of IL-1, IL-6 and TNF-α in the gingival crevicular fluid are known to contribute to acute and chronic inflammation in periodontal disease [17–20]. In the current study, IL-6 was significantly different between days 0 and 6 in severe group. Overall, the concentrations of some inflammatory biomarkers were lower at day 6 when compared with baseline values in the severe group showing that inflammation somehow subsided days after dental extractions. However, postoperative pain scores remained high in this group when compared with those with minimal disease at day 6 showing that the association between pain scoring and inflammatory biomarkers is not clear. Meanwhile, other cytokines (i.e. SCF) were also able to differentiate the two groups at baseline, while some (i.e. sFAS, IL-6, SDF-1, MCP-1) were significantly associated with dental parameters (i.e. number of teeth resorption, missing teeth and teeth fracture). These biomarkers could be possibly associated with the pathogenesis of oral disease and further investigation is warranted. A current study in our laboratory is investigating the relationship between local (i.e. tissue biopsy) and serum inflammatory biomarkers in cats with severe periodontal disease.

One potential limitation of this study was the lack of randomization and the use of a scoring system to determine group disease severity. The allocation of cats was ultimately based on the number of dental extractions. However, the scoring system was able to differentiate the two groups based on severity particularly when prevalence of rescue analgesia is considered. Additionally, age, body weight, anesthesia and surgery times were significantly different between groups. Indeed, age and body weight were previously correlated with the severity of periodontal disease [32]. These factors (i.e. age, body weight, anesthesia and surgery) might have biased pain scoring by the observer and limited the findings of this study since one could infer disease severity based on surgery and anesthesia times. On the other hand, the authors improved the robustness of the study design by including objective outcome measures such as food intake and the evaluation of serum inflammatory cytokines.

## Conclusion

This study showed that severe oral disease and multiple dental extractions produce severe pain and inflammation that require long-term analgesic treatment. Opioids were often required for up to 2 days after surgery. This condition affects food intake with an ultimate consequence for the welfare and nutritional status of these patients. Pain scores and inflammatory biomarkers were associated with dental parameters and could predict postoperative analgesic requirements. The concentrations of serum inflammatory biomarkers after dental extractions and between severity groups were described and could provide future insights into the pathogenesis of oral disease in cats.

## Supporting information

S1 File Raw data. (XLSX)
